# Multicenter Clinical Validation of a Cartridge-Based Real-Time PCR System for Detection of Coccidioides spp. in Lower Respiratory Specimens

**DOI:** 10.1128/JCM.01277-17

**Published:** 2018-01-24

**Authors:** Michael A. Saubolle, Bette R. Wojack, Anne M. Wertheimer, Atehkeng Z. Fuayagem, Stephen Young, Brian A. Koeneman

**Affiliations:** a Laboratory Sciences of Arizona/Sonora Quest Laboratories, Banner University Medical Center-Phoenix, University of Arizona, Phoenix, Arizona, USA; b The BIO5 Institute and the Applied Biosciences Graduate Interdisciplinary Program, University of Arizona, Tucson, Arizona, USA; c Division of Geriatrics General Internal Medicine and Palliative Medicine, University of Arizona, Tucson, Arizona, USA; d TriCore Reference Laboratories, Albuquerque, New Mexico, USA; e Laboratory Sciences of Arizona/Sonora Quest Laboratories, Tempe, Arizona, USA

**Keywords:** coccidioidomycosis, Coccidioides spp., valley fever, PCR, fungal diagnosis

## Abstract

Available methods for the diagnosis of coccidioidomycosis have significant shortcomings relative to accuracy and timeliness. We retrospectively and prospectively evaluated the diagnostic performance and reproducibility of a new cartridge-based real-time PCR assay for Coccidioides spp. directly in lower respiratory secretions and compared them to today's “gold standard,” fungal culture. The GeneSTAT Coccidioides assay uses a 106-bp target sequence repeated multiple times (∼60×) per genome, thus lowering the limit of detection (LOD) for extracted DNA to 10 genome equivalents/ml. A total of 332 prospective and retrospective individual patient specimens were tested. The retrospective samples consisted of 100 bronchoalveolar lavage or bronchial wash (BAL/BW) (51 positive and 49 negative by culture) specimens that had been collected previously and stored at −70°C. These samples were tested by the GeneSTAT Coccidioides assay across three clinical test sites. The sensitivity was 100%, and the specificity ranged between 93.8% and 100%. There was minimal variance in the percent agreement across the three sites, 95.6% to 100%. Additionally, a total of 232 fresh (prospective) deidentified BAL/BW specimens were tested across the three clinical sites, which included a number of specimens from Southern California to provide a diversity of isolates. Specimens were tested by fungal culture, with any isolates of Coccidioides, except for one, being confirmed by molecular means (AccuProbe). The sensitivity of the GeneSTAT Coccidioides assay across the three sites was 100% (4/4) for positive fresh specimens, and the overall specificity of the assay was 99.6% (227/228), ranging from 98.1% to 100%. In testing for cross-reactivity, the assay was 100% specific when screened against 47 different bacterial, viral, and fungal species.

## INTRODUCTION

Coccidioidomycosis (valley fever) is caused by the dimorphic fungal genus Coccidioides, which contains two species, Coccidioides immitis and C. posadasii. Although coccidioidomycosis is endemic to the Southwest United States, as well as portions of Mexico and South and Central America, the range of C. immitis is restricted to Southern California (being most concentrated in the San Joaquin Valley) and, as recently determined, an area in inland Washington State (1–3). In contrast, C. posadasii is found in the other regions where the disease is endemic, including south and central Arizona, where the majority of coccidioidomycosis cases are identified. There have been no significant differences identified in the clinical presentations in humans between the two species (1, 2).

Airborne arthroconidia of Coccidioides spp. enter the body via the respiratory route, usually causing self-limited pulmonary infections in humans and other vertebrate hosts. Clinical presentations can be quite variable. About one-third of all infections are symptomatic and lead infected individuals to seek medical attention. The most common presentation in humans is that of a community-acquired pneumonia (CAP), with Valdivia et al. reporting a high prevalence (29%) of CAP cases in a southern AZ study being due to a coccidioidal infection detected serologically (4–6). Only 3 to 5% of overall cases disseminate to other body sites, including the skin, brain, bone, and meninges, when diagnosis and treatment are delayed (1). The disseminated form of coccidioidomycosis is often severe and can result in patient death. There is a continued increase in the number of recognized cases of coccidioidomycosis within the areas in the United States where the disease is endemic. In Arizona there are an estimated 150,000 infections annually resulting in 1,735 hospitalizations. Hospitalization costs total approximately $86,000,000 (or $50,000 per hospitalization) (7–9). In addition to the health care costs, there are significant productivity costs associated with valley fever given its debilitating nature. In cases where infection is resolved, patients usually acquire a specific and lifelong immunity to the fungus unless previously infected persons become immunocompromised (1, 7, 9).

Currently available diagnostic methods for coccidioidomycosis have significant shortcomings relative to accuracy and timeliness, resulting in an average time to diagnosis of 5 months following the first health care visit and 1 month of lost work time (5). This level of missed diagnosis or misdiagnosis leads to ineffective use of antimicrobial agents for extended periods, which can, in turn, lead to extended illness, increased morbidity and mortality, prolonged patient suffering, and higher treatment costs.

Although the Arizona Department of Health Services recommends that all patients presenting with symptoms of CAP be tested for coccidioidomycosis, current estimates are that only approximately 12% of cases are tested (5). One reported reason for such a low testing rate is clinicians' lack of confidence in serologic studies, which, due to their potential for false-positive/negative results and patients' slow humoral antibody response, result in nonactionable information (5, 7, 9).

Currently used diagnostic methods include histopathology, fluorescence microscopy using calcofluor white potassium hydroxide wet preparations, fungal culture, and serologic evaluation. Serology, the most commonly used test method, is problematic in that the humoral antibody response is delayed after infection, leading to potential false negatives. False-positive results may also occur, especially with IgM-directed enzyme immunoassay tests. Fungal cultures present additional problems, including poor sensitivity and frequent delay in recovery, with an average of 4 days to recognition and, at times, requiring 3 or more weeks (2, 10). Additionally, working with Coccidioides in the laboratory is hazardous to laboratory staff, requiring biosafety level 3 precautions (1).

Binnicker et al. (11) reported on a laboratory-developed test (LDT) based on real-time PCR which is commercially available only through the Mayo Clinic Reference Laboratory (Rochester, MN). Mitchell et al. (12) also reported on a real-time PCR assay developed for the BD MAX system (Becton Dickinson, Sparks, MD). The BD MAX test is an LDT not approved by the FDA, and therefore, it might not be practical for the routine microbiology lab. Rapid detection of Coccidioides in patient specimens can provide benefits to both patients and at-risk laboratorians. Patients can benefit through early diagnosis resulting in appropriate intervention and avoidance of inappropriate antibiotics and costly additional testing; laboratorians benefit by reduction of the potential risk of exposure to the growing fungal culture and a decrease in time to answer.

The objective and design of this study were to evaluate the diagnostic performance and reproducibility of the GeneSTAT Coccidioides assay on the GeneSTAT instrument (DxNA LLC, St. George, UT) compared to those of the current “gold standard,” fungal culture and, except for one isolate, confirmation by GenProbe AccuProbe Coccidioides immitis Culture Confirm assay (which detects both C. immitis and C. posadasii; Hologic, Inc., San Diego, CA).

## MATERIALS AND METHODS

### Patient population and testing site characteristics.

Patient samples were tested at three sites in the southwestern United States (Laboratory Sciences of Arizona/Sonora Quest Laboratories, Tempe AZ; the BIO5 Institute, University of Arizona, Tucson, AZ; and TriCore Reference Laboratories, Albuquerque NM). Although the GeneSTAT Coccidioides assay does not differentiate between C. immitis and C. posadasii, one of the testing laboratories also received specimens from central California to add to the geographic diversity of specimens. There were 9 specimens submitted from CA, of which 2 were positive for Coccidioides spp. and 7 negative. Although the Coccidioides isolates were not identified to the species level, both C. immitis and C. posadasii were members of the analytical limit of detection (LOD) and inclusivity studies and were shown to be reactive.

All sites received IRB approval (Western Investigational Review Board, Puyallup, WA; study numbers 1155878, 1158542, and HRRC 15-154) prior to the start of the study. This study was performed to generate comparative data to be used in the FDA submission for clearance of the assay, instrument platform, and PCReports software (GeneSTAT system).

### Method comparison.

The study design consisted of testing a cohort of retrospective specimens as well as a cohort of prospective specimens. Due to the low prevalence of culture-proven coccidioidomycosis, stored frozen retrospective samples were tested to demonstrate the test system's sensitivity and fresh refrigerated prospective samples were used to demonstrate product specificity.

Both groups consisted of remnant deidentified bronchoalveolar lavage (BAL) or bronchial wash (BW) specimens and, per FDA guidance, no informed consent was required. Specimens in both the retrospective and prospective cohorts were deidentified relative to the culture result if available prior to PCR testing.

### Retrospective specimen testing.

Stored deidentified BAL/BW specimens (minimum of 3 ml) were maintained by each collection site at −70°C or below. Specimens included previously confirmed samples negative and positive for Coccidioides infection by culture, with identities of isolates confirmed by AccuProbe. At the time of processing, a thawed sample was aliquoted (1 ml) into 3 microcentrifuge tubes. Two were maintained at the test laboratory, and the third was immediately shipped to a nonprofit genomics laboratory for PCR screening using their in-house validated PCR assay and bidirectional sequencing (Translational Genomics Research Institute [TGen], Flagstaff, AZ). Only samples that had their culture-based infection status confirmed by the reference PCR assay were included in the study for testing by the GeneSTAT Coccidioides assay on the GeneSTAT instrument.

### Prospective specimen testing.

BAL/BW specimens submitted for routine fungal culture from all study sites were deidentified, and aliquots (minimum of 3 ml) were prepared for the study. All positive specimens required positive cultures with identities of isolates confirmed by AccuProbe. Reported positive and negative samples were tested by the GeneSTAT Coccidioides assay on the GeneSTAT instrument. Performance of the GeneSTAT Coccidioides assay was compared to culture/AccuProbe results, except for one isolate which was presumptively identified by morphological characteristics and clinical infection confirmed by serologic means in both serum and cerebrospinal fluid (CSF); discordant results were confirmed with the validated reference PCR method and bidirectional sequencing performed by TGen, the third-party laboratory contracted for this study.

### Inclusion criteria.

Inclusion criteria for retrospective specimens included the following: a minimum 3-ml sample volume, confirmation of negativity or positivity by fungal culture/AccuProbe and TGen PCR assay, a duration not more than 3 days after treatment with Sputolysin reagent (EMD MilliporeSigma, Darmstadt, Germany), and previous maintenance at −70°C or below. Inclusion criteria for the prospective specimens included the following: a duration not more than 7 days after sample collection, storage at 2 to 8°C, and never having been frozen.

### Culture methods.

Cultures were performed as per laboratory operating procedure at each individual study site; all study sites followed standard culture procedures and were accredited by the College of American Pathologists. Only sites where isolates of Coccidioides spp. were routinely confirmed using AccuProbe were selected to participate in the study. One site did not confirm an isolate by AccuProbe but rather identified it presumptively based on morphology since serologic data on serum and CSF showed increasing anti-Coccidioides antibody, confirming the clinical diagnosis of coccidioidomycosis.

### Molecular methods.

The GeneSTAT Coccidioides assay is a qualitative real-time PCR-based assay that detects Coccidioides target DNA that has been extracted from BAL or BW samples. The Coccidioides-specific PCR assay targets a 106-bp sequence that is present in multiple copies (∼60) within a genus-specific transposon genome of both C. posadasii and C. immitis. The assay was developed at TGen and is exclusively licensed to DxNA for implementation in its cartridge-based system using the GeneSTAT system (13). The target sequence of the assays was selected based on its high number of repeats, sensitivity, and specificity in a region identified in the NCBI database as a “copia-like retrotransposon.” The details of the assay design, including the primer and probe sequences, are published elsewhere (13). The manual cells lysis and DNA extraction methods were also developed at TGen (14; J. Bowers, E. Driebe, J. Nibecker, N. Ampel, S. Hoover, J. Galgiani, B. Wojack, M. Saubolle, P. Keim, and D. M. Engelthaler, presented at the Coccidioides Study Group Conference, Surprise, AZ, 25 March 2010).

### Sample preparation and DNA extraction (retrospective and prospective specimens).

While the GeneSTAT Coccidioides assay and instrument are designed to be a sample-to-result system, given the refractory nature of the Coccidioides endospores/spherules, specimens must be treated and DNA extracted prior to placement in the assay cartridge. BAL/BW specimens were maintained at 2 to 8°C and processed at least through the Sputolysin digestion stage within 7 days of collection. Once specimens had undergone Sputolysin digestion, they were maintained at 2 to 8°C and were processed through the DNA extraction procedure in 3 days or less. Extracted DNA samples were stored for up to 8 h at 2 to 8°C or frozen at −20°C or lower for up to 30 days prior to testing. A 1-ml aliquot of BAL/BW specimen and 1 ml of external positive and negative controls were first treated with 200 μl of Sputolysin. The mixture was vortexed for 10 s and incubated for 20 to 25 min at room temperature. The treated specimen was then centrifuged at 2,000 × *g* for 20 min. The supernatant was pipetted off and discarded, taking care not to disturb the pellet. The supplied lyticase was rehydrated according to the assay instructions, and 180 μl of the lyticase solution was added to the BAL/BW samples and the two external controls. Each pellet was then resuspended by gently pipetting the sample up and down until the pellet was completely resuspended in the lyticase solution. The sample was then placed in a dry-block heat bath set at 37°C and incubated for 30 to 35 min. The sample was then allowed to cool and briefly centrifuged for 5 to 10 s to bring the contents to the bottom of the tube. DNA extraction was performed using the Qiagen QIAamp DSP DNA minikit (Qiagen BioSciences, Germantown, MD) according to the package insert, with the exception of loading only 120 μl of elution buffer to the spin column.

### Amplification and detection.

One hundred microliters of the extracted DNA is placed inside a labeled sample vial, which is then attached to the Coccidioides assay cartridge. The single-use cartridge contains all the necessary reagents for amplification and detection of the Coccidioides target DNA as well as a human DNA control sequence that is used as an internal control to monitor the presence of inhibitors in the PCR and to ensure that the sample preparation process is adequate. All information to run the assay as well as lot number and expiration date is contained on the radio frequency identification (RFID) tag on the cartridge. The cartridge contains two PCR wells. One well contains PCR reagents for amplification and detection of Coccidioides DNA and the human gene internal control (reaction well 1). The second well contains only the reagents for amplification/detection of Coccidioides DNA (reaction well 2). The cartridge is placed in the GeneSTAT instrument, the RFID tag is read by the instrument, the user follows a series of data entry steps, including adding sample ID information, and the run is started. All subsequent steps of the assay process are performed by the GeneSTAT instrument without user intervention, i.e., transfer of sample from the sample vial to the reaction wells in the cartridge, hydration of PCR reagents, PCR amplification, and real-time detection of target sequences and result analysis. The entire GeneSTAT system process, from cartridge loading to the result, takes approximately 1.5 h to complete. External positive and negative controls were run each day and on each new lot of cartridges. The run was started within 15 min of the transfer per assay requirement. If the extracted DNA sample was not going to be run within 8 h, it was stored at −70°C or below for not more than 30 days. GeneSTAT instruments were connected to a single laptop computer running PCR Reports software, version 3.1.103.1.

### Analysis of discrepant results.

Specimens producing discrepant results between the culture and GeneSTAT Coccidioides assay in the prospective section of the study were tested further using a different validated PCR assay (TGen reference assay) and bidirectional sequencing to resolve the discrepancy. The TGen reference assay was a completely independent PCR assay targeting the internal transcribed spacer (ITS) region, whereas the GeneSTAT assay targets a genus-specific transposon genome, the specifics of which have been published elsewhere (15).

### Reproducibility study.

A panel of samples was prepared by the sponsor for this study using dilutions of cultures of spherules/endospores. The reproducibility panel consisted of nine members, blind and randomized to the operator. The panel included the following prepared samples: negative (BAL), 3 replicates; low positive (C. posadasii: 1× LOD in BAL), 3 replicates; and medium positive (C. posadasii: 3× LOD in BAL), 3 replicates. Two operators performed reproducibility testing at each of three study sites. Each operator tested the reproducibility panel once a day over a period of 5 nonconsecutive days. Each operator tested a specific pair of GeneSTAT instruments throughout this study (panel members were split among the instruments [i.e., four samples on one and five on the other]). Four GeneSTAT instruments were used at each site for this testing. Each study site was provided a different lot of GeneSTAT Coccidioides assay reagents and cartridges for this testing.

Each member of the reproducibility panel was confirmed by the GeneSTAT Coccidioides assay prior to shipment to the sites. Each day, a reproducibility panel was extracted in one batch along with external negative and positive quality controls. The extracted panel and controls were tested by each operator, being split among their assigned pair of GeneSTAT instruments. Therefore, 11 samples (9 panel members plus a positive and negative control) were extracted and tested among two instruments per operator each day.

## RESULTS

### Cross-reactivity and LOD.

The LOD for extracted DNA from both strains was 10 genome equivalents/ml. In testing for cross-reactivity, the assay was reportedly 100% specific when screened against 47 different bacterial, viral, and fungal species (see the supplemental material).

### Retrospective specimens.

A total of 100 BAL/BW specimens, 51 positive and 49 negative, stored at −70°C and meeting the study inclusion criteria were tested across the three clinical test sites. Specimens included previously confirmed negative and positive samples for Coccidioides infection from fungal culture and confirmed by culture; specimens positive for Coccidioides infection were further confirmed by a DNA probe assay (AccuProbe). The sensitivity and specificity for the GeneSTAT Coccidioides assay compared to culture and AccuProbe confirmation are shown in Table 1. The sensitivity was 100% across the three sites, and the specificity ranged between 93.8% and 100%. There was little variance in the agreement across the three sites, 95.6% to 100%. Two false-positive results were obtained from the GeneSTAT Coccidioides assay. Both samples (TC-1205 and TC-1250) were called as positives in the assay due to the generation of weak signals in one of the two PCR wells in the GeneSTAT cartridge. The threshold cycle (*C_T_*) values were 40.6 (TC-1205) and 44.8 (TC-1250). The root cause(s) for the results is not known, although possible causes are (i) low-level contamination of the samples with the Coccidioides PCR template from an unknown source and (ii) true positive results generated by low levels of Coccidioides in the samples that were not detected by culture or the reference PCR method.

**TABLE 1:** Retrospective sample testing summary

Reference testing^*a*^ result, all sites	No. with indicated GeneSTAT result		Total	Sensitivity (%)	Specificity (%)^*b*^
	Positive	Negative			
Positive	51	0	51	100.00	
Negative	2	47	49		95.90
Total	53	47	100		

a Reference testing was done by culture.

b Specificity between the testing sites ranged from 93.80% to 100.00%.

### Prospective specimens.

A total of 232 fresh (prospective) deidentified BAL/BW specimens meeting the inclusion criteria were tested at the three clinical sites. Nine of the specimens were collected from a health care facility in Southern California (Kaiser Permanente, Southern California) and were sent to one of the clinical sites for testing in order to expand the geographic diversity of the tested specimens. Samples were tested by fungal culture, and except for one isolate, culture-positive samples were confirmed by AccuProbe. The sensitivity and specificity for the GeneSTAT Coccidioides assay compared to those of culture and AccuProbe are shown in Table 2. The sensitivity of the GeneSTAT Coccidioides assay across the three sites was 100% (4/4) for fresh specimens, and the overall specificity of the assay was 99.6% (227/228), ranging from 98.0% to 100%.

**TABLE 2:** Prospective sample testing summary

Reference testing^*a*^ result, all sites	No. with indicated GeneSTAT result		Total	Sensitivity (%)	Specificity (%)^*b*^
	Positive	Negative			
Positive	4	0	4	100.00	
Negative	1	227	228		99.60
Total	5	227	232		

a Reference testing was done by culture.

b Specificity across testing sites ranged from 98.00% to 100%.

One false-positive result was obtained from the GeneSTAT Coccidioides assay. The sample was called positive by the assay and was negative by fungal culture. Further analysis of this sample by an independent validated PCR method with bidirectional sequencing confirmed the fungal culture result.

The definitive reason for this false-positive result is not known; however, subsequent analysis of the raw fluorescence data from the test cartridge indicated that there may have been inefficient rehydration of the lyophilized master mix in the PCR wells in the cartridge at the beginning of the assay process. The cause of the putative rehydration problem is not known; however, this was the only sample in the entire method comparison of 332 prospective and retrospective samples for which this phenomenon was observed.

### Reproducibility testing.

Reproducibility testing was performed at three sites, with two different operators at each site. A total of 90 BAL specimens each that were at 1× LOD, at 3× LOD, and negative were tested as part of the study. A summary of test results for each site individually and as a total is shown in Table 3. The expected result, 100% agreement, was produced by the GeneSTAT Coccidioides assay for all samples producing a valid result. There were a total of three invalid results produced from two of the testing sites, two invalid results at site number 1 and one invalid result at site number 2. These invalid samples were not retested. Percent agreement was calculated using only those samples that produced a valid result.

**TABLE 3:** Reproducibility study summary

Coccidioides challenge sample reactivity	% Agreement (no. of samples with expected result/total) [no. of invalid runs]			
	Testing site 1	Testing site 2	Testing site 3	Testing sites combined
Coccidioides at 3× LOD in BAL	100.0 (29/29) [1]	100.0 (30/30) [0]	100.0 (30/30) [0]	100.0 (89/89) [1]
Coccidioides at 1× LOD in BAL	100.0 (30/30) [0]	100.0 (30/30) [0]	100.0 (30/30) [0]	100.0 (90/90) [0]
Coccidioides-negative BAL	100.0 (29/29) [1]	100.0 (30/30) [0]	100.0 (29/29) [1]	100.0 (88/88) [2]

## DISCUSSION

With the increasing incidence of coccidioidomycosis within and outside the region where the disease is endemic, having a rapid and definitive diagnostic tool becomes ever more important. The GeneSTAT Coccidioides assay and GeneSTAT instrument, which processes one specimen at a time, were developed to provide a rapid, definitive molecular diagnostic tool that could be implemented broadly once regulatory clearance is received. While there are two other molecular assays for Coccidioides currently available (11, 12), both are laboratory-developed tests (LDTs) that are not FDA cleared and thus may not be approved for reimbursement by Medicare. While the LDT assays have not been compared directly to the GeneSTAT Coccidioides assay, based on reports in the literature for these LDT assays (11, 12), the limit of detection for the GeneSTAT assay would appear to be similar. However, based on the assay design, the GeneSTAT assay may be more specific than the assays of Binnicker et al. and Mitchell et al., because the latter two targeted the multicopy ITS2 region (spacer unit within the rRNA gene) shared by all fungi. On the other hand, the GeneSTAT assay was designed to hit a Coccidioides-specific target, rather than a pan-fungal target like the ITS region. TGen also originally conducted *in silico* (BLAST) validation of the assay by searching the retrotransposon target against the entire NCBI genome database, with hits only to the Coccidioides genome. DxNA further performed *in silico* analysis of primers/probes, and no cross-reactivity was predicted (D. Engelthaler and M. Wood, personal communication).

While the GeneSTAT instrument and assay cartridge were designed for “sample in, result out” testing, the refractory nature of the Coccidioides endospores/spherules necessitated a pretreatment and extraction in order to ensure that DNA from endospores and spherules in the specimen was fully released. This has been a common theme with the other molecular assays that have been reported, where aggressive sample preparation techniques have been required to break down the spherules (11, 12; Bowers et al., presented at the Coccidioides Study Group Conference, Surprise, AZ, 25 March 2010).

In summary, sensitivity and specificity results in this study have shown that the GeneSTAT Coccidioides assay performed on the GeneSTAT instrument is an accurate diagnostic test compared to the current gold standard of fungal culture with DNA probe confirmation of positive results. The 100% reproducibility across multiple operators at multiple sites confirms the robustness of the assay and consistency of assay results, including at the level of the LOD of the assay. This real-time PCR assay can provide results in a matter of hours, as opposed to up to weeks for a fungal culture. Through the availability of this rapid and definitive information, false negatives that can be seen with serology and the result delay associated with culture can be avoided and appropriate intervention can be initiated earlier.

The full role of the GeneSTAT Coccidioides assay in the diagnosis of coccidioidomycosis is to be further elucidated, since the FDA submission used culture of the organism alone as a gold standard. The full extent of the assay's performance in other clinical situations, such as culture negativity but serologically, histopathologically, and/or clinically diagnosed coccidioidomycosis, remains to be seen. The present submission to the FDA included only BAL and BW specimens to minimize review complexity; future studies will be required to validate the use of the assay for other specimen types, such as sputum and CSF. Additional specimen type submissions would be submitted to the FDA for approval. Still, the use of BAL and BW specimens could further improve clinicians' ability to diagnose the disease earlier in the disease process, thereby improving patient care as well as lowering costs of treatment through earlier intervention. The assay has been FDA cleared and is available to provide a rapid diagnosis method for identifying patients infected with Coccidioides spp. at any laboratory capable of performing high-complexity molecular testing. Further FDA clearance of additional clinical specimens at a later date would enhance the diagnostic capabilities of the assay and of Medicare reimbursement of such testing, as well.

## Supplementary Material

Supplemental material
