# Supplementary material for: Multicenter Clinical Validation of a Cartridge-Based Real-Time PCR System for Detection of Coccidioides spp. in Lower Respiratory Specimens
Source: J Clin Microbiol. 2018 Jan 24;56(2):e01277-17. doi: 10.1128/JCM.01277-17 (PMC5786707; doi:10.1128/JCM.01277-17)
Supplement: Supplemental material [file supp_56_2_e01277-17__index.html]

Supplemental material 

# Multicenter Clinical Validation of a Cartridge-Based Real-Time PCR System for Detection of Coccidioides spp. in Lower Respiratory Specimens

## Supplemental material

- Supplemental file 1 -

  Table S1 (List of bacterial, fungal, and viral strains against which GeneSTAT *Coccidioides* assay was tested without any cross-reactivity being evident)

  XLSX, 11K
